# Understanding the Usefulness of Self-Escape Technologies in Underground Mining: Perspectives of Metal/Nonmetal Miners

**DOI:** 10.1007/s42461-025-01368-0

**Published:** 2025-09-26

**Authors:** Eugene A. Gyawu, Kwame Awuah-Offei, D. A. Baker

**Affiliations:** 1 Mining Department, Missouri University of Science and Technology, Rolla, MO USA; 2 Human Systems Integration Branch, Pittsburgh Mining Research Division, National Institute for Occupational Safety and Health, Pittsburgh, PA USA

**Keywords:** Mine safety, Human factors, Self-escape, Human systems integration, Human-centered designs, Underground metal and nonmetal mines

## Abstract

Research on mine self-escape often focuses on coal mining, while perspectives from underground metal/nonmetal miners remain understudied despite their distinct emergency response challenges and unique operating environments. Using a scenario-based survey approach, this study evaluated underground metal/nonmetal miners’ perceptions of the usefulness of 18 hypothetical self-escape interventions and how these perceptions are influenced by worker characteristics. Employment type was the strongest predictor of usefulness ratings, with hourly employees rating several self-escape interventions significantly higher than salaried employees, including those related to improving self-contained self-rescuers (SCSRs) and tethered guidance systems. The data suggested potential trends where perceived usefulness increased with more time spent underground and decreased with higher education levels. While previous research found no relationship between these characteristics and coal miners’ perceptions of self-escape technologies, our findings suggest these factors do influence metal/nonmetal miners’ views. This work contributes to the broader understanding of human systems integration in mining self-escape, reinforcing the notion that technology priorities can differ by user groups, while acknowledging that certain core safety needs may transcend these differences.

## Introduction

Self-escape in the context of a mine emergency is defined as “the ability of an individual or group of miners to remove themselves from the mine using available resources” (National Research Council (NRC), 2013). While “available resources” encompasses many life-saving self-escape technologies, the mining industry continues to face challenges in ensuring that miners can effectively use such resources during emergencies [5, 13, 21]. Though stakeholders have called for self-escape technologies that are designed to account for user group differences to enhance usability, increase adoption rates, and improve overall safety and efficiency in high-risk environments like mining [18], very little exists in the literature that uses empirical, human systems integration to evaluate the effect of user groups on the perceived usability and effectiveness of self-escape interventions. The literature also lacks data from miners that elicit their preferences and input for self-escape interventions. The few papers that exist [8, 16] tend to focus on the views of coal miners. Admittedly, the major mining safety incidents requiring self-escape tend to occur in coal mining. However, metal/nonmetal mining also has its own unique hazards that could precipitate events requiring self-escape. For example, between 2018 and 2022, there were more than 11,000 accidents reported in metal and nonmetal and stone, sand, and gravel, with nearly 1000 of those attributed to ground fall, explosive events, or fire [17].

In a previous study, Gyawu et al. [8] *coal* miners’ feedback on 21 proposed technological interventions to improve their self-escape knowledge, skills, and abilities (KSAs). Using a scenario-based survey approach, the study evaluated the perceived usefulness of these interventions in relation to user group characteristics among coal miners only. The study found that overall, coal miners ranked interventions related to self-contained self-rescuers (SCSRs) and refuge alternatives (RAs) as the most useful; however, counter to expectations, user group variables did not significantly impact the perceived usefulness of the interventions. Instead, the study found that a miner’s decision about using an RA was the strongest predictor of usefulness, with those opting to shelter in the RA rating most interventions significantly more useful than those opting to self-escape.

Similar to the previous study conducted for underground coal miners, this research aims to assess the perceived usefulness of self-escape interventions among underground metal/nonmetal miners. In line with human systems integration approaches, this study also examines whether these perceptions vary as a function of demographic and user group variables. Using a scenario-based survey approach, the study (1) evaluates miners’ perceptions of 18 hypothetical self-escape interventions (customized for metal/nonmetal underground mining) and (2) analyzes the influence of demographic and user group variables on these perceptions, including employment type, education level, work group, work location, work schedule, and time spent underground. Additionally, we assess miners’ decisions to shelter in place during an underground roof fall emergency in relation to self-escape interventions.

The novelty of this work is that it uniquely focuses on gathering feedback from underground metal/nonmetal miners regarding self-escape interventions, an area that has received less attention compared to coal mining. Previous work by Gyawu et al. [8] evaluated self-escape interventions in coal mining, particularly in fire scenarios. However, metal/nonmetal mining presents distinct challenges, requiring a different approach to evaluate self-escape strategies. The mining environment, safety hazards, and self-escape technologies differ significantly between these sectors. For example, coal mining is more vulnerable to gas-related dangers such as methane and explosions [3], whereas metal/nonmetal mining faces issues related to ground stability and heat stress, especially in deeper mining operations [4]. Therefore, this research addresses an important gap by eliciting insights directly from metal/nonmetal miners about their perceptions of the usefulness of self-escape interventions.

This study also aims to provide insights on how these perceptions vary based on user groups’ variables (e.g., work role, employment status) that impact miners’ tasks and responsibilities. Understanding how perceptions about self-escape interventions vary with user groups is critical, as these interventions are only effective if accepted and properly used by the right group of miners who will need the intervention most in an emergency. While managers and regulators decide which interventions are developed and implemented, the on-the-ground use during emergencies is largely in the hands of rank-and-file miners. If rank-and-file miners do not view these interventions as useful, there is a risk that they will not be used effectively during an emergency, potentially compromising safety outcomes [8]. To explore these perceptions, this study employs a scenario centered on a roof-fall emergency, a more probable hazard in underground metal/nonmetal mining. The insights gathered are expected to inform the design and implementation of more effective, human-centered self-escape interventions tailored to the unique challenges of hard rock mining.

The final objective of this study is to broadly compare the perspectives of metal/nonmetal miners with those of coal miners, as explored in the authors’ previous work [8]. Although the interventions and the scenarios used in the two studies are slightly different, there is substantial overlap in the methodology that warrants comparison. Understanding these differences as well as differences in hazard prevalence can shed further light on tailoring self-escape interventions to the specific needs of each mining sector.

## Methods

### Overview

**Fig. 1:**
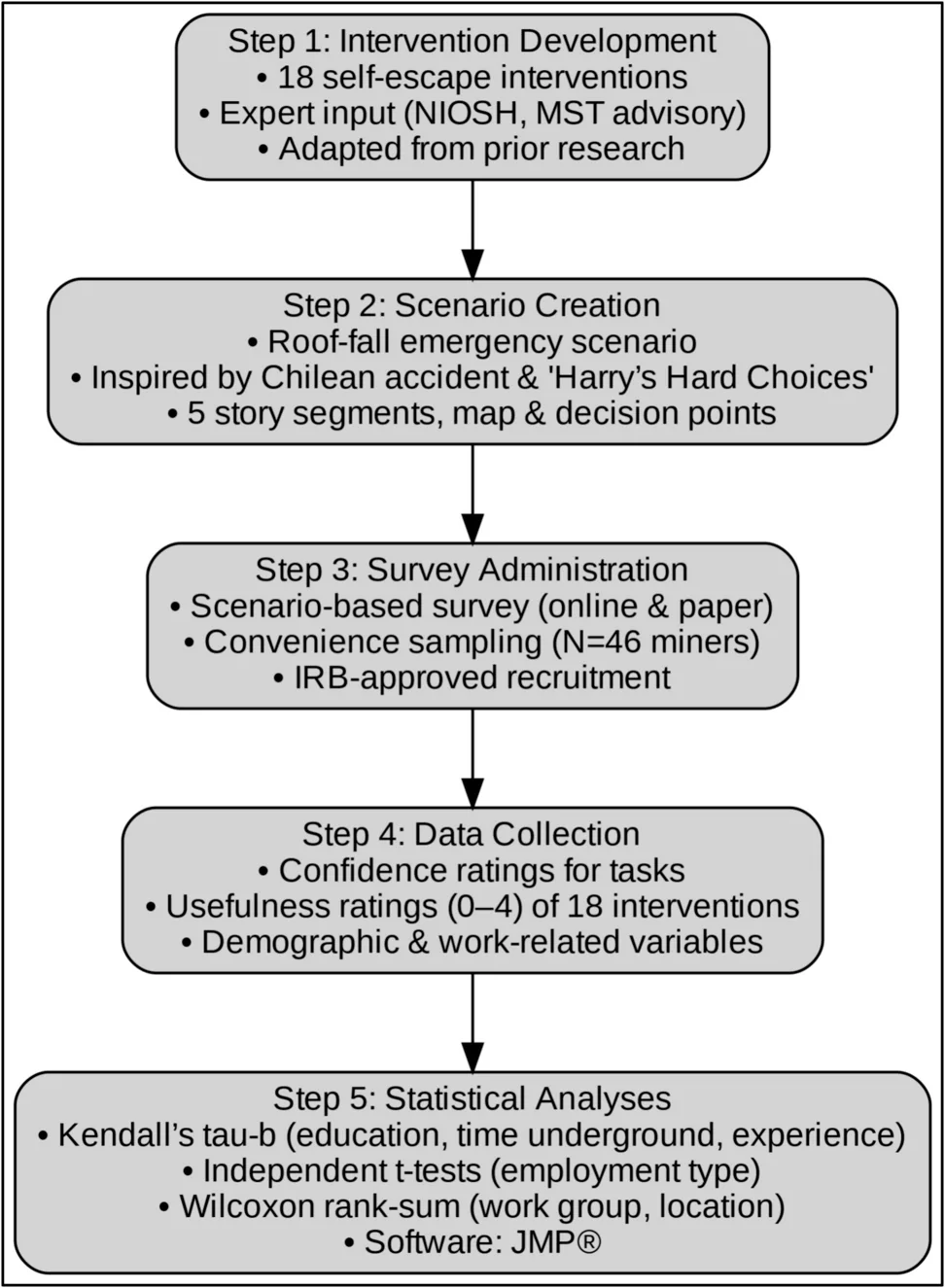
Methodological flow of the study evaluating perceptions of self-escape interventions among underground metal/nonmetal miners

A scenario-based survey was conducted, using both online and paper and pencil methods to increase our reach, targeting miners from US metal/nonmetal mines. Participants rated the perceived usefulness of 18 hypothetical self-escape interventions designed to support critical self-escape tasks outlined in the scenario. These ratings were analyzed across various common demographic indicators to determine if perceptions differed based on user-group identity. Figure 1 shows a visual flowchart of the methodology section of this study.

### Participants

Approval was received for this study from the University of Missouri Institutional Review Board (MU IRB). Participants were recruited from multiple underground metal and nonmetal mines in the USA via convenience sampling, utilizing word-of-mouth, email invitations sent to mine supervisors, and referrals. Forty-six participants completed the survey. Of these, approximately 88% responded male (39), 5% responded female (2), and 7% did not provide male or female indication. The high proportion of males mirrors what is typically observed in the US mining industry (US Census, 2022; McWilliams et al. [14]). All participants were over the age of 18, with 62% between the ages of 25 and 54 and 9% over 55. In terms of educational level, 32% of participants had a high school diploma (14), 11% held a trade or associate degree (5), 36% had a bachelor’s degree (16), 16% earned a master’s degree (7), and 5% obtained a Ph.D. (2).

### Scenario

Instead of presenting participants with an uncontextualized list of 18 hypothetical self-escape interventions, a scenario-based survey design was utilized, embedding these interventions within the context of an underground mining emergency resulting from a roof fall. This approach was adopted because, first, research indicates that realistic scenario-based surveys more effectively assess an individual’s behavior during emergency decision-making [11]. Second, since our focus was on interventions designed to assist with low-confidence self-escape tasks identified by NIOSH [16], the scenario-based design allowed us to orient participants’ mindsets around these tasks. This was crucial because performing self-escape tasks is rarely required in practice and low usefulness ratings could stem from the participants’ difficulty envisioning relevant tasks rather than a genuine assessment of the intervention’s effectiveness. By structuring the scenario in a way that required participants to engage with these tasks, the aim is to eliminate this potential bias. Also, participants assessed their own confidence in completing these tasks before evaluating the interventions. Finally, the scenario-based survey design aligns with human-centered design principles, emphasizing the evaluation of interventions within its intended work environment [7].

The scenario was inspired by the 2010 Chilean mining accident [23] and the CDC-NIOSH training module “Harry’s Hard Choices” [20]. The original “Harry’s Hard Choices” exercise immerses trainees in a detailed, hypothetical coal mine emergency, requiring them to choose appropriate safety protocols at different decision points. Our interdisciplinary research team and industry stakeholders modified this scenario to a roof fall in a hard rock mine and related questions to align with our project needs. The emergency details were adjusted to primarily cover low-confidence KSAs identified by NIOSH [16]. Additionally, the questions were reframed so that participants rated their confidence in performing various tasks rather than selecting which tasks to perform. After indicating their confidence levels, participants were asked to rate the usefulness of a subset of real or hypothetical interventions that could plausibly assist them in completing these tasks.

The final scenario included an introduction with brief instructions, character descriptions, and an overview of the mine (along with a map available for review). The introduction was followed by five interconnected story segments. Table 1 provides an overview of each segment, the targeted competencies, and the specific intervention assessed. The reader can find the full survey at http://mining-sustainability.com/wp-content/uploads/2024/08/metal_and_non-metal_survey.pdf.

**Table 1:** Overview of the scenario plot and targeted task competencies for each story segment and the corresponding list of interventions presented to participants after each story segment. The shortened codes used in the analyses are included for reference. The technology readiness level (TRL) and status of the state of the technology are also presented along with the code

Summary of story part	Target task competencies	Presented interventions	Analyses code/*TRL (status)
Part 1-Emergency onset. Cracking sound from the roof, crew contact the control room	Know the ERP for roof falls, gather critical miner details, communicate effectively, and assess roof conditions	Reference card with simplified step-by-step instructions from ERP on how to respond in a roof fall situation and emergency radio channels that you can refer to at any time	Communication_1/Existing (standard practice)
		Reference card listing what critical information about the miners to gather and who to report it to	Communication_2/Existing (paper-based tools used in industry)
		A robot with the capability of detecting slight ground movement and inspecting and reporting poor roof conditions	Robot_1/Conceptual (not used in mining yet)
Part 2-Emergency alarm sounds, fall of heavy debris, miners covered in clouds of fine dust	Knowing what alarms mean, properly don an SCSR and communicate with SCSR on	Emergency alarms with audible voice notifications	Communication_3/Piloted (being trialed in industry)
		An SCSR that is easier to assemble and don than current technology	SCSR_1/Piloted (Ongoing R&D)
		An SCSR that allows you to talk while wearing	SCSR_2/Conceptual (prototype stage)
Part 3-Escape progress slow, visibility is low, mine truck has an accident	Using tether/tagline, switching over SCSR’s	Tether/taglines that allow one miner to disconnect and reconnect easily without breaking entire connection	Tether_1/Existing (modification of current systems)
		Lifeline with voice commands explaining each symbol as you touch/squeeze them	Lifeline_1/Conceptual (prototype idea)
		Hand-held guidance system that provides ongoing audio and text updates about current safe evacuation route including when refuge alternative is the best option	Map_1/Conceptual (cross-industry inspiration, not in mining)
		Improved SCSRs that allow miners to switchover without breathing contaminated air	SCSR_3/Piloted (Under development)
Part 4-Some miners can’t continue, a refuge alternative (RA) is nearby	Knowing when to use an RA, activating RA and knowing information to gather and report to command center	Refuge alternative with fewer required steps for entering	RA_1/Piloted (design refinements tested)
		Clearly labelled instructions inside shelter with step-by-step instructions for activating an in-place refuge alternative	RA_2/Existing (used in training modules)
		Reference card listing what critical information about the situation to gather and who to report it to	Communication_4/Existing (checklist-based tools)
Part 5-An injured miner in the RA is feeling well enough to leave, communication between the RA and surface is lost	Escape option-Route planning, map reading	Interactive mine maps with highly reflective signage under low-lighting conditions	Map_3/Piloted (in limited contexts)
		Active voice/text turn-by-turn guidance based on the safest route at the moment through the mine similar to “vehicle GPS”	Map_4/Conceptual (prototype idea)
	Stay option-RA maintenance, reestablishing communication, listening and maintaining awareness	Improved and simplified way of purging bad air and setting up scrubbers	RA_3/Piloted (Industry R&D)
		Robot that can be deployed from refuge alternative to troubleshoot and re-establish communications	Robot_2/Conceptual (prototype ideas)
		Display in the refuge alternative that shows the current location of other miners, and air and temperature monitoring data directly outside of the RA	Map_2/Conceptual (not yet implemented)

*TRL Technology readiness level)

The interventions presented in Table 1 represent a mix of technologies currently used in underground mining, those under development or being piloted, and purely conceptual ideas adapted from analogous industries. For example, interventions related to self-contained self-rescuers (SCSR_1, SCSR_2, SCSR_3) and step-by-step refuge alternative instructions (RA_2) are grounded in existing or actively piloted technologies within mining operations. Similarly, tether/tagline systems (Tether_1) and improved refuge alternative entry designs (RA_1) reflect modifications of established practices. In contrast, other interventions such as robotic systems for roof monitoring or communication restoration (Robot_1, Robot_2), turn-by-turn interactive evacuation guidance (Map_4), and dynamic displays of environmental conditions from within a refuge alternative (Map_2) are more speculative, designed to test miners’ openness to novel solutions. By including both current and conceptual tools, the study sought to capture not only perceptions of familiar, incremental improvements but also miners’ potential receptivity to emerging innovations that may shape the future of self-escape technologies.

Similar to the earlier study by Gyawu et in the final segment of the story (part 5), participants encountered a situation where an injured miner forced to remain in a refuge alternative (RA) because of injury begins to feel well enough to consider self-escape, but when they try to contact the command center, they find they cannot establish communication. Participants were asked to imagine themselves in this situation and then indicate whether they would choose to stay in the RA or attempt to self-escape. Based on their decision, participants were then prompted to answer questions regarding tasks and interventions related to their choice. After completing these questions, participants were asked to consider the tasks and interventions associated with the alternative option. This scenario design served two key purposes: first, it allowed us to collect comprehensive feedback on both RA-related and self-escape interventions from all participants. Second, and more importantly, by presenting a situation where staying in the RA was clearly safer than attempting self-escape, we could identify participants who rejected RAs regardless of circumstances. Previous industry feedback had indicated that some miners fundamentally oppose using RAs, which could lead them to negatively rate all RA-related interventions regardless of merit. This approach affords a distinction between critiques based on specific intervention features versus those stemming from a potential anti-RA stance.

### Measures

The primary variables of interest in this study are the perceived usefulness ratings of 18 self-escape interventions (Table 1), the decision to either leave or stay in a refuge alternative, and self-reported user group variables (Table 2). The interventions, which are either hypothetical or conceptual, were designed to aid in completing the critical self-escape tasks as identified by NIOSH [16]. The list of interventions was compiled by examining those used in other industries or by mining industries outside the USA and was refined through consultations with mining technology and safety experts, NIOSH’s Escape, Rescue, and Training Team (Pittsburg Mining Research Division) and the Mine Escape Research, Innovation, and Technology Center advisory board at Missouri University of Science and Technology.

**Table 2:** Responses for each user group variable (employment type, time spent underground, work group, work experience, employment type, and work location)

User group variable	*N*	%
Employment type		
Hourly	16	35.56
Salaried	27	60.00
Prefer not to say	2*	4.44
Total	45	100
Time spent underground		
0%	5	11.11
25%	14	31.11
50%	9	20.00
75%	10	22.22
100%	7	15.56
Total	45	100
Work group		
Production	18	40.91
Maintenance	6	13.64
Safety	6	13.64
Engineering	9	20.45
Other	5	11.36
Total	44	100
Work experience		
Less than 1 year	2*	4.55
1–5 years	14	31.82
6–10 years	10	22.73
11–15 years	5	11.36
16–20 years	4	9.09
More than 20 years	9	20.45
Total	44	100
Work location		
Working face	14	30.43
Outby	3	6.52
Surface	14	30.43
Other	13	28.26
Total	44	100

*Not included in analyses due to sample size

Following a similar approach to our previous study [8], several employment-related demographic variables were captured to examine differences across user groups with respect to the perceived usefulness of the 18 interventions. A core principle of human systems integration is recognizing that people have unique needs and characteristics that influence how they interact with systems [10]. While it may not be feasible to address every individual’s unique needs, grouping users based on characteristics such as experience, work role, education level, and physical or cognitive attributes allows for more targeted analysis aligned with specific design goals [12].

Variables that are likely to influence miners’ perceptions of self-escape interventions were included. Work group and work location were included, given previous studies have shown these variables can affect miners’ safety attitudes and behaviors [1, 15]. In underground metal/nonmetal mining, the nature of a miner’s work (e.g., production, maintenance) and the location of that work (underground working face, surface) significantly affects their exposure to emergency scenarios, and thus their perceptions of the utility of self-escape interventions may differ depending on whether they are regularly in more hazardous zones such as the working face.

Time spent underground is a crucial variable to consider when evaluating the effectiveness of self-escape interventions in metal/nonmetal mining operations. Miners who spend more time underground are exposed to a greater number of hazardous situations and have more direct experience with emergency situations [6, 9]. As a result, their feedback on self-escape interventions may reflect practical, hands-on insights into how these interventions would perform under real conditions. This variable is particularly significant because it can influence miners’ familiarity with specific escape protocols and interventions and their ability to respond quickly and effectively during an emergency [15].

Research has also shown that more experienced miners tend to have higher confidence in their ability to handle emergency situations, including the use of self-escape devices [8]. Experienced miners may also have encountered more varied emergency situations, providing them with a broader understanding of potential risks and how to mitigate them [10, 12]. This experience might influence their perceptions of the usefulness and effectiveness of self-escape interventions, as they may be more critical of interventions that do not meet their practical needs [1]. Education is included for similar reasons. Previous research suggests that education level positively correlates with adoption and use of new technologies [2, 22], which could lead to important differences in perceptions of proposed safety interventions. Examining whether differences in education level impact miners’ perceptions of proposed safety interventions will help further contextualize any observed differences.

By considering these variables, the study aims to capture a comprehensive view of how different user groups within the metal/nonmetal mining workforce perceive self-escape interventions, allowing for more targeted and effective design and implementation strategies. This approach follows the human-centered design principle of recognizing and addressing the diverse needs and attitudes of end-users [10]. Future interventions can be better tailored to the practical realities miners face, based on their specific work conditions.

### Procedure

The procedure for this study closely mirrored that of the underground coal mining study described in [8]. Participants were presented with a scenario-based survey, where they moved through a hypothetical roof fall mining emergency scenario divided into five segments. Each segment corresponded to critical self-escape tasks relevant to that stage of the emergency, allowing participants to reflect on their knowledge, skills, and abilities (KSAs) while also evaluating the usefulness of various proposed self-escape interventions related to the task(s) at hand.

However, unlike the coal mining study, this metal/nonmetal mining study offered participants two modes of participation: an online version, administered through QualtricsXM, and a paper-based version, available for those with limited Internet access or who preferred a traditional format. Both formats followed the same structure and content, ensuring consistency across responses.

Upon accessing the survey (either online or via paper), participants were first presented with a cover letter outlining their rights, privacy protections, and the study’s objectives. After agreeing to participate, they were given an overview of the study procedure, introduced to the mining scenario, and provided with a description of the mine and a map they could refer to at any time. Participants progressed through the scenario at their own pace, responding to two sets of questions after each segment.

In the first set, they rated their confidence in performing 3–5 critical self-escape tasks using a slider scale from “0” (no confidence) to “10” (extremely confident). The second set asked them to evaluate the usefulness of 3–5 hypothetical interventions designed to assist in those tasks, with responses rated on a slider scale from “0” (not at all useful) to “4” extremely useful). After completing the fifth segment, participants were asked to decide whether they would choose to shelter-in-place in a refuge alternative with an injured miner or attempt self-escape and were then prompted to assess tasks and interventions related to their decision. Participants provided demographic information and self-identified user group variables after the scenario-based questions.

### Statistical Analysis

JMP® software was used to analyze usefulness ratings of the 18 interventions and differences in these ratings across the user-group variables shown in Table 2. Several statistical analyses were used to examine the relationships between user group variables and the perceived usefulness of 18 self-escape interventions. For each user group variable, appropriate statistical tests were selected to evaluate the significance of the findings.

Kendall’s tau-b was used to analyze the effect of education level, time spent underground, and work experience on the usefulness ratings. This non-parametric test was chosen because it assesses the strength, direction, and significance of relationships between ordinal variables and a continuous outcome, such as perceived usefulness, and is appropriate for small or uneven group sizes. To compare differences in perceived usefulness based on employment type (a nominal variable), independent samples *t*-tests were conducted, applying Welch’s correction when Levene’s test indicated the equal variance assumption was violated. For work location and work group, which are nominal variables with more than two levels, Wilcoxon rank-sum tests were applied. This test was used because it compares the distributions of usefulness ratings between groups, making it suitable for non-parametric data where normality assumptions may not hold.

Four variables, the decision to stay or leave the RA, work schedule, sex, and age, were excluded from the analysis due to highly unequal sample sizes. For the decision to stay or leave, 41 participants indicated they would stay, while only 5 participants indicated they would leave. Similarly, for work schedule, 40 selected “set schedule,” while only 4 selected “rotates.” Although the findings provide useful insights into miner perceptions of self-escape interventions, the sample used is not statistically representative of the broader underground metal/nonmetal mining workforce. Convenience sampling without probabilistic control or stratification limits the generalizability of the results. Therefore, the findings should be interpreted as exploratory and hypothesis-generating rather than conclusive for the industry as a whole.

## Results

### Overall Usefulness Ratings for Interventions

**Fig. 2:**
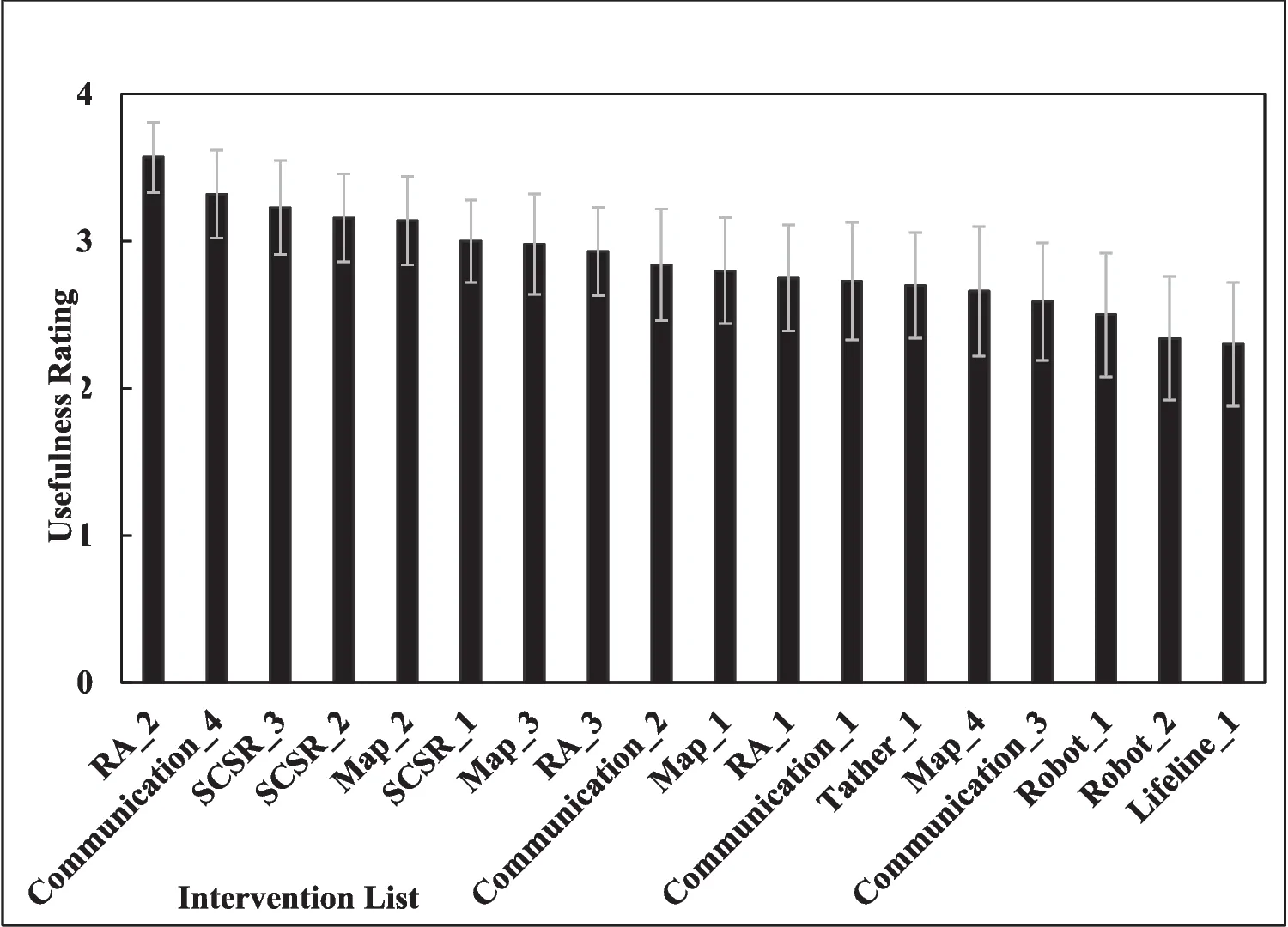
Mean usefulness rating of all 18 interventions, arranged in decreasing order (*N* = 46). Error bars represent two times the standard error

Each of the 18 self-escape interventions described in Table 1 were rated on a scale of 0 to 4, where higher values indicated greater perceived usefulness. Figure 2 shows the mean usefulness ratings for each intervention across all participants. Ratings fell approximately between 1.88 and 3.38; that is, from slight to moderately useful to very useful. Improvements related to self-contained self-rescuers (SCSRs), refuge alternatives (RAs), and communication dominated the top five interventions that miners perceived as the most useful; however, sections 3.2 to 3.7 illustrate that degree of usefulness and order of top priorities can differ as a function of certain user-group variables.

### Effect of Education Level

Kendall’s tau-b was used to evaluate the strength, direction, and significance of the relation between education level and usefulness rating of each of the 18 interventions. However, to control for multiple comparisons, a Bonferroni correction was applied to the significance threshold. This correction was calculated by dividing the conventional alpha level (0.05) by the number of tests (18), resulting in an adjusted significance threshold of 0.0028. Results, shown in Table 3, revealed no significant correlations when evaluating each test at the adjusted threshold. However, looking at the data more holistically, it is important to note that nearly all tests show a negative correlation direction (as education increases, perceived usefulness tends to decrease); several of these show a moderate or strong effect size, and many of those are significant at the conventional alpha level of 0.05. Given the sample size was somewhat small and the number of tests high, it may be worth considering that there is an underlying negative correlation between education level and perceived usefulness that could be more evident in a larger sample size.

**Table 3:** Overall usefulness ratings for the 18 technologies and Kendall’s tau-b correlation coefficients for education level, time spent underground, and work experience

Intervention list	Education (*N* = 44)		Time spent underground (*N* = 44)		Work experience (*N* = 44)	
	(τb)	*p* (two-tailed)	(τb)	*p* (two-tailed)	(τb)	*p*(two-tailed)
Communication_1	−0.05	0.682	0.07	0.601	−0.37	0.003*
Communication_2	−0.15	0.232	0.07	0.599	−0.01	0.956
Communication_3	−0.35	0.006*	0.31	0.013*	−0.17	0.177
Communication_4	−0.18	0.178	0.15	0.247	−0.09	0.489
Lifeline_1	−0.31	0.015*	0.33	0.008*	−0.27	0.031*
Map_1	−0.05	0.698	0.09	0.459	−0.19	0.121
Map_2	0.11	0.419	−0.08	0.539	−0.19	0.139
Map_3 (a)	−0.10	0.437	0.22	0.091	−0.19	0.138
Map_4 (a)	−0.18	0.166	0.16	0.215	−0.15	0.223
RA_1	−0.22	0.082	0.21	0.088	0.02	0.896
RA_3	−0.01	0.946	0.15	0.242	−0.06	0.673
RA_2	−0.07	0.603	0.00	0.980	−0.01	0.947
Robot_1	−0.19	0.129	0.21	0.098	−0.27	0.031*
Robot_2	−0.10	0.446	0.16	0.182	−0.20	0.106
SCSR_1	−0.25	0.058	0.27	0.037*	−0.09	0.471
SCSR_2 (a)	−0.24	0.072	0.38	0.003*	−0.06	0.652
SCSR_3 (a)	−0.27	0.038*	0.36	0.005*	−0.09	0.489
Tether_1	−0.30	0.017*	0.31	0.014*	−0.16	0.208

Very weak: *τ* < 0.10

Weak: 0.10 < *τ* < 0.19

Moderate: 0.20 < *τ* < 0.29

Strong: *τ* > 0.30

*t-test is significant at the 0.05 level (two-tailed)

### Effect of Time Spent Underground

To investigate the relation between time spent underground and perceived usefulness, once again Kendall’s tau-b was used and an adjusted significance threshold of 0.0028. Results, shown in Table 3, revealed no significant correlations when evaluating each test at the adjusted threshold. However, it is important to consider the data from a broader perspective. Nearly all tests indicate a positive correlation, showing that as time spent underground increases, perceived usefulness tends to increase. Several of these correlations exhibit moderate to strong effect sizes, and many are significant at the conventional alpha level of 0.05. These findings suggest that there is an underlying positive correlation between time spent underground and perceived usefulness that could be more evident in a larger sample size.

### Effect of Work Experience

The relation between work experience and usefulness was also evaluated using Kendall’s tau-b and the adjusted significance threshold of 0.0028. As with the previous two analyses, no intervention demonstrated a statistically significant correlation with work experience at the adjusted threshold. However, in this case, looking at the data more holistically provides little evidence of an underlying relationship. While nearly all tests show a negative correlation direction, most are weak and non-significant even at the conventional alpha level of 0.05.

### Employment Type Analysis

A series of independent sample *t*-tests were conducted to examine differences in usefulness ratings between hourly and salaried employees for the 18 self-escape interventions. Using the adjusted significance threshold of 0.0028 as previously described, the results, shown in Table 4, revealed significant differences in the perceived usefulness of several interventions. The data suggests an underlying pattern. Hourly employees rated the usefulness of the technologies higher than salaried employees, with seven of those being significant at the 0.0028 threshold and an additional four being significant at the conventional 0.05. For the ratings that are significantly different, the effect sizes were large (0.8 to 1.2) or very large (> 1.2).

**Table 4:** Independent samples *t*-tests comparing hourly and salaried worker’s usefulness ratings for the 18 technologies, ordered by hourly workers’ mean rating, highest to lowest

Intervention list	Hourly M (SD)	Salaried M (SD)	*t*	df	*p*	Cohen’s d
SCSR_3 (a)	3.81 (0.40)	2.92 (1.13)	−3.66	34	< 0.001**	0.96
SCSR_2 (a)	3.75 (0.58)	2.88 (1.03)	−3.48	40	< 0.001**	0.98
RA_2	3.63 (0.62)	3.50 (0.91)	−0.53	39	0.599	0.16
Tether_1	3.63 (0.62)	2.12 (1.11)	−5.66	40	< 0.001**	1.58
Communication_4 (a)	3.63 (0.50)	3.23 (1.03)	−1.66	38	0.106	0.46
Map_3 (a)	3.63 (0.50)	2.73 (1.15)	−3.47	37	< 0.001**	0.94
SCSR_1	3.62 (0.50)	2.73 (0.92)	−4.08	40	< 0.001**	1.13
Communication_3 (a)	3.56 (0.63)	2.12 (1.37)	−4.66	38	< 0.001**	1.25
Map_4	3.44 (0.73)	2.27 (1.59)	−3.24	38	0.003*	0.88
RA_3	3.38 (0.72)	2.69 (0.97)	−2.61	38	0.013*	0.78
RA_1	3.38 (0.72)	2.46 (1.24)	−3.02	40	0.004*	0.86
Map_2	3.25 (0.86)	3.04 (1.11)	−0.69	38	0.493	0.21
Communication_1 (a)	3.25 (0.77)	2.54 (1.44)	−2.07	39	0.045*	0.58
Communication_2	3.19 (1.11)	2.69 (1.23)	−1.35	34	0.186	0.42
Map_1	3.19 (1.11)	2.65 (1.23)	−1.45	34	0.156	0.46
Lifeline_1	3.19 (1.11)	1.88 (1.24)	−3.53	35	< 0.001**	1.10
Robot_1	2.94 (1.48)	2.27 (1.34)	−1.47	29	0.152	0.48
Robot_2	2.88 (1.31)	2.08 (1.38)	−1.88	33	0.069	0.59

(a) indicates significant Levene’s test; result reported is for equal variances not assumed

*t-test is significant at the 0.05 level (two-tailed)

**t-test is significant at the 0.0028 level (two-tailed)

The overall mean rating and standard deviation for hourly workers were 3.44 (SD = 0.76), while for salaried workers, the overall mean rating and standard deviation were 2.67 (SD = 1.18). All SCSR interventions (SCSR_1, SCSR_2, and SCSR_3) were rated significantly higher by hourly workers compared to salaried workers (all *p’s* < 0.001). For example, SCSR_3 was rated 3.81 by hourly workers but only 2.92 by salaried workers. Hourly workers rated RA interventions higher, with significant differences observed in RA_3 and RA_1; however, both groups rated RA_2 (clearer step-by-step instructions to deploy an RA) among the most useful interventions.

Overall, the findings suggest that hourly employees rated the interventions as more useful than salaried employees, with several interventions showing large and significant differences. These results highlight the need for considering employment type when evaluating the perceived usefulness of self-escape interventions for underground metal/nonmetal miners.

### Work Location and Work Group Analysis

A series of Wilcoxon rank-sum tests were performed to assess differences in usefulness ratings for 18 interventions across different work locations. None of the differences were statistically significant after the Bonferroni correction (*p* = 0.0028) nor did any meet the conventional alpha level of 0.05. The analysis was repeated using the work group variable and produced the same result. The findings suggest perceived usefulness of the interventions did not vary as a function of work location or work group.

## Discussion

### Perspectives of Metal and Nonmetal Miners

This study contributes to a growing body of research aimed at improving the safety and effectiveness of self-escape interventions [8, 16, 18]. The results of this study provide valuable insights into the perceptions of self-escape interventions among different user groups in the metal and nonmetal mining sectors.

A key finding from the study is that employment type significantly influenced the perceived usefulness of self-escape interventions, with hourly employees consistently rating interventions as more useful than salaried employees. This was particularly evident in the stronger preferences for interventions such as Tether_1, SCSR_1, and Communication_3, which showed large effect sizes (Cohen’s *d* = 1.58, 1.13, and 1.25, respectively). These results suggest that hourly employees may hold different beliefs about what tools should be developed for self-escape interventions to address their unique needs. In mining, duties for hourly workers involve significant physical labor and exposure to hazardous conditions, and previous research has documented the need for safety interventions specific to their work environments [10, 15]. The large differences in perceived usefulness could suggest hourly workers have different attitudes toward new technology adoption or that they perceive their risk levels differently. While daily hands-on experiences could inform hourly workers’ assessment of intervention usefulness, salaried employees often have more administrative duties [19], which could lead them to consider the potential administrative burdens or costs associated with implementing a new technology. In this case, broader experience dealing with the challenges of technology selection and implementation could lead to more cautious ratings. Future research may help clarify the underlying factors driving the differing perceptions between hourly and salaried employees.

The results regarding education level and time spent underground are also interesting. Although no significant correlations were found after applying the Bonferroni correction, the consistent directionality and presence of significant differences at the conventional alpha level are worth considering. For instance, there is a general trend of negative correlation between education level and perceived usefulness; miners with higher levels of education tended to rate interventions lower. This relationship is consistent with literature indicating that although individuals with higher education may be more open to adopting new technology, they may scrutinize the complexity and usability of safety technologies more [10, 22]. Conversely, there is a general trend of positive correlation between time spent underground and perceived usefulness, that is, miners who spend more time underground tended to rate interventions higher. This aligns with prior research that suggests greater exposure to underground conditions increases miners’ appreciation of practical, real-world interventions [15].

The results of this study indicate that employment type, education level, and time spent underground were associated with different perceptions of self-escape interventions in underground metal and nonmetal mining. While other variables examined (work experience, work location, and work group) did not show significant differences in our sample, these factors may warrant further investigation with larger sample sizes or in different contexts. Additional research could help validate the trends observed here and expand our understanding of how different miners perceive and interact with self-escape interventions.

This study adds to the literature on human systems integration in mining self-escape by documenting how perceptions of interventions varied across different demographic groups. The findings suggest that user characteristics may be important to consider when evaluating self-escape interventions. Future research could investigate whether tailoring interventions to specific user groups affects their implementation or effectiveness.

The metal and nonmetal mining industry may benefit from considering these demographic factors when selecting and implementing self-escape interventions. Further research, including practical trials, would help determine how to best incorporate these findings into intervention development and implementation to enhance the effectiveness of self-escape interventions.

### Comparison with Previous Research on Underground Coal Mining

A secondary objective of this work was to broadly compare the perspectives of metal and nonmetal miners with those of coal miners from these authors’ previous work [8]. This was an effort to evaluate whether metal and nonmetal miners’ perspectives on interventions to improve self-escape competence differ from those of coal miners. It is important to note that this comparison can only be qualitative because of the differences in the scenarios and interventions.

In the coal mining study [8], approximately 48% (56) of participants chose to stay in the RA with an injured miner, while 52% (60) opted to try to self-escape, even knowing communication with the command center was lost. However, in the present study, 91% (41) of the participants chose to stay in the RA, while only 0.8% (4) opted to try to self-escape. This could suggest that underground metal and nonmetal miners have a higher trust in the safety and efficacy of RAs, possibly due to training, prior experiences, or the specific context of their work environment. However, it is also possible that the nature of the emergency and the mine type (roof fall vs. fire; hard rock vs. coal) may be what is driving differences in decisions to use an RA. In this case, differences in RA decisions between the two studies would be explained by the context of the scenario rather than reflecting inherent differences between coal and metal/nonmetal miners. Future research could help shed light on how emergency types and contexts affect RA decisions, such as whether coal miners are more likely to use an RA during roof falls than fires and whether metal/nonmetal miners are less likely to use an RA during fires than roof falls.

The coal mining study found no significant relationship between perceived usefulness ratings and user group variables such as employment type, workgroup, experience, schedule, and location, while on the other hand, the present study found several of the user group variables were related to usefulness ratings. Interestingly, the only strong predictor of usefulness ratings for the 21 interventions included in the coal mining study was the decision to stay in the RA, with higher ratings coming from those who chose to stay; the predictive value of the stay/escape decision could not be evaluated in the metal nonmetal study because nearly all participants chose to stay.

When looking at the overall ratings, regardless of user group variables, the coal and metal/nonmetal studies share similarities in miners’ perceptions of the usefulness of certain interventions. These include interventions related to improving SCSRs and RAs. For example, interventions that would make SCSRs easier to don and would allow verbal communication were rated among the highest in both studies. This was also the case for providing clearly labeled instructions for deploying an RA, which was also rated high in both studies regardless of user group. Across both studies and all user groups, interventions focused on improving SCSR usability and RA deployment emerged as consistently high priorities.

## Conclusion

The work assessed underground metal/nonmetal miners’ feedback on 18 interventions that could increase confidence in performing critical self-escape tasks and compared responses across numerous user group variables. It also offers a broad qualitative comparison between results of the present study and a similar study conducted with underground coal miners.

The present study accomplished this through a scenario-based survey administered to miners from the US underground metal/nonmetal industry, asking respondents to rate the perceived usefulness of 18 hypothetical interventions that could potentially aid with completion of critical self-escape tasks.

The findings revealed that employment type significantly influenced intervention ratings, with hourly employees rating certain interventions as more useful compared to salaried employees. While some variables like education level and time spent underground showed potentially meaningful correlations that warrant further investigation, other variables such as work group, work location, and work experience did not show significant differences in this sample.

The qualitative comparison between metal/nonmetal miners and coal miners revealed differences in the relationship between user groups variables and perceived usefulness, but also revealed shared priorities across both sectors, particularly regarding SCSR and RA improvements. Future research could continue to build on these insights through similar survey methodologies targeting rank-and-file miners and further exploration of user group factors in larger and more diverse samples. These patterns, however, should be interpreted cautiously. Given the exploratory design, non-probabilistic sampling, and modest sample size, the results may not generalize to the broader metal/nonmetal mining workforce. Instead, the study should be viewed as hypothesis-generating, highlighting areas where further investigation with larger, stratified samples may be warranted.

This work contributes to the broader understanding of human systems integration in mining self-escape, reinforcing the notion that perception of technology can differ by user groups, while acknowledging that certain core safety needs and priorities may transcend these differences across the mining industry.

## Data Availability

The data that support the findings of this study are openly available at https://nam02.safelinks.protection.outlook.com/?url=http%3A%2F%2Fmining-sustainability.com%2Fwpcontent%2Fuploads%2F2024%2F08%2F116_Data_Interventions_and_Demographics.xlsx&data=05%7C02%7Ceagnvh%40mst.edu%7Cc9371db4a3c14e564fcc08dcc3884a51%7Ce3f.
